# The differences in cytokine signatures between severe fever with thrombocytopenia syndrome (SFTS) and hemorrhagic fever with renal syndrome (HFRS)

**DOI:** 10.1128/jvi.00786-24

**Published:** 2024-06-25

**Authors:** Zishuai Liu, Xiaoyu Xue, Shuying Geng, Zhouling Jiang, Ziruo Ge, Chenxi Zhao, Yanli Xu, Xiaolei Wang, Wei Zhang, Ling Lin, Zhihai Chen

**Affiliations:** 1 Department of Infectious Disease, Beijing Ditan Hospital, Capital Medical University, Beijing, China; 2 Department of Infectious Disease, Beijing Ditan Hospital, Peking University, Beijing, China; 3 Department of Infectious Diseases, Yantai Qishan Hospital, Yantai, China; Lerner Research Institute, Cleveland Clinic, Cleveland, Ohio, USA

**Keywords:** cytokines, differential diagnosis, hemorrhagic fever with renal syndrome, severe fever with thrombocytopenia syndrome

## Abstract

Severe fever with thrombocytopenia syndrome (SFTS) virus and hantavirus are categorized under the Bunyavirales order. The severe disease progression in both SFTS and hemorrhagic fever with renal syndrome (HFRS) is associated with cytokine storms. This study aimed to explore the differences in cytokine profiles and immune responses between the two diseases. A cross-sectional, single-center study involved 100 participants, comprising 46 SFTS patients, 48 HFRS patients, and 6 healthy controls. The study employed the Luminex cytokine detection platform to measure 48 cytokines. The differences in cytokine profiles and immune characteristics between the two diseases were further analyzed using multiple linear regression, principal component analysis, and random forest method. Among the 48 cytokines tested, 30 showed elevated levels in SFTS and/or HFRS compared to the healthy control group. Furthermore, there were 19 cytokines that exhibited significant differences between SFTS and HFRS. Random forest analysis suggested that TRAIL and CTACK were predictive of SFTS, while IL2Ralpha, MIG, IL-8, IFNalpha2, HGF, SCF, MCP-3, and PDGFBB were more common with HFRS. It was further verified by the receiver operating characteristic with area under the curve >0.8 and *P*-values <0.05, except for TRAIL. Significant differences were observed in the cytokine profiles of SFTS and HFRS, with TRAIL, IL2Ralpha, MIG, and IL-8 being the top 4 cytokines that most clearly distinguished the two diseases.

**IMPORTANCE:**

SFTS and HFRS differ in terms of cytokine immune characteristics. TRAIL, IL-2Ralpha, MIG, and IL-8 were the top 4 that differed markedly between SFTS and HFRS.

## INTRODUCTION

Severe fever with thrombocytopenia syndrome (SFTS) is a viral hemorrhagic fever (VHF) caused by a novel bunyavirus called SFTS virus (SFTSV) transmitted by ticks. It was first discovered in rural areas of central China in 2010 (1). The mortality rate associated with SFTS is extremely high, ranging from 12% to 30% in previous cases in China, South Korea, and Japan (2, 3). As of 2019, there have been 12,000 confirmed cases of SFTS in China with a mortality rate remaining as high as 7% (4). Studies have shown that neurological manifestations were more frequent in patients with severe SFTS than those with generalized types, with these neurological manifestations often predicting a poorer prognosis (5).

The pathogenic mechanism of severe fever with thrombocytopenia syndrome is not entirely understood at present. However, research suggests that immune dysfunction and cytokine storm play a crucial role in the mortality of patients (6, 7). During the acute phase of SFTS infection, Zhang et al. confirmed that SFTS induces an immune response in monocytes, leading to the activation of STAT1 and the differentiation of macrophages into the M1 phenotype. It results in the production of pro-inflammatory cytokines such as tumor necrosis factor-α (TNF-α), interleukin-1β (IL-1β), and IL-6, which ultimately lead to tissue damage (8). Additionally, monocytes themselves undergo severe apoptosis and necrosis (9). Studies have shown that in SFTS patients who died, there were elevated levels of cytokines such as IL-1ra, IL-6, IL-10, G-CSF, IP-10, MCP-1, IL-1, IL-8, MIP-α, and MIP-1β during the acute phase (7). However, SFTSV infected directly the central nervous system, leading to frequently detect in CSF from patients of SFTS-associated encephalopathy/encephalitis with elevated MCP-1 and lL-8 (10).

Another important VHF is HFRS caused by hantavirus. HFRS shares similarities in epidemiology, seasonality, and clinical manifestations with SFTS, making it difficult to distinguish between the two diseases (11, 12, 13). For example, patients with HFRS can develop central nervous system complications such as encephalitis and toxic encephalopathy (14). Research has suggested that cytokine storm is also the mechanism for HFRS (15). Pro-inflammatory cytokines, such as tumor necrosis factor-α, interferon-α (IFN-a), interleukin-1β, IL-6, and IL-8, also increase significantly during the acute phase of HFRS and are correlated with disease severity (16). Studies have shown that TRAIL-dependent apoptosis can inhibit the replication of hantavirus (17) and that increased levels of IL-6 and IL-15 are associated with the risk of severe disease in HFRS (18, 19), suggesting that targeting IL-6, IL-15, and TRAIL may be important and novel therapeutic targets for HFRS (19).

Although there have been studies on the differences in clinical manifestations between the two diseases, there is currently no research comparing the cytokine profiles of SFTS and HFRS. Therefore, our study aimed to identify differences in cytokines between the two diseases by comparing the levels of cytokines in the acute phase of SFTS and HFRS. This may guide the exploration of potential treatment targets and provide insights into pathogenic mechanisms.

## MATERIALS AND METHODS

### Research design

This study is a cross-sectional, single-center research that randomly enrolled 94 patients treated at Yantai Qishan Hospital from June to December 2022. The patient group consisted of 46 SFTS patients and 48 HFRS patients who were matched for disease severity and selected for generalized patients, as well as 6 healthy controls (HCs) matched to the patient group for gender and age. Patients with generalized SFTS meet the following diagnostic criteria (20): (i) epidemiological history, (ii) most commonly seen in middle-aged and older people with a temperature of 38°C–39°C; clinical symptoms include general malaise, muscle aches, and obvious gastrointestinal symptoms such as nausea, vomiting, diarrhea, and so on; (iii) the patients’ results of positive SFTSV nucleic acid quantitative RT-qPCR in acute-phase patient (duration of illness 5–11 days) were acquired from medical records, which were conducted on plasma samples by clinician using the Nucleic Acid Extraction and Purification Kit and the Fluorescence PCR Kit for SFTSV RNA (Sun Yat-Sen University Daan Gene Co., Ltd, Guangzhou, China). Patients with moderate HFRS fulfill the following diagnostic criteria (21): (i) epidemiological history, (ii) body temperature of 39°C–40°C; the following clinical signs: bulbar conjunctival edema, marked skin and mucous membranes ecchymosis, oliguria, hypotension, or shock tendency; (iii) positive serum-specific IgM antibodies of Hantaan virus causing hemorrhagic fever with renal syndrome. The exclusion criteria were as follows: (i) presence of other viral infections, (ii) autoimmune diseases, (iii) hematological diseases, (iv) patients with tumors undergoing radiotherapy and chemotherapy, (v) blood transfusions within the past 2 weeks, (vi) chronic kidney disease, and (vii) refusal to provide informed consent.

### Sample collection and cytokine measurement

The demographic baseline information, clinical symptoms and signs, and laboratory tests of the patients were collected. Anticoagulated blood was collected from the patients within 12 hours of admission before any therapeutic interventions, and the plasma was separated and frozen at −80°C for cytokine detection. The Luminex cytokine detection platform was used, with the main experimental material being Bio-Rad Bio-Plex Pro Human Cytokine Screening 48-Plex Panel (Cat. No. 12007283). A total of 48 cytokines were analyzed, including IL-1α, IL-1β, IL-1ra, IL-2, IL-2Rα, IL-3, IL-4, IL-5, IL-6, IL-7, IL-8, IL-9, IL-10, IL-12 (p40), IL-12 (p70), IL-13, IL-15, IL-17, IL-16, IL-18, LIF, G-CSF, GM-CSF, M-CSF, SCF, SCGF-β, IFN-γ, IFN-a2, TNF-α, TNF-β, TRAIL, Basic FGF, VEGF, PDGF-BB, HGF, β-NGF, SDF-1α, MIG, IP-10, MCP-1, MIP-1α, MIP-1β, MCP-3, RANTES, Eotaxin, CTACK, GRO-α, and MIF.

### Definition

The study defined neurological signs as the presence of at least one of the following changes: muscle tension, involuntary movement, and abnormal reflexes. Consciousness disorders manifested as drowsiness, confusion, lethargy, and delirium, with severe cases leading to coma. The study used activated partial thromboplastin time (APTT) prolongation in the intrinsic coagulation pathway as the standard for prolonged coagulation time.

### Statistical analysis

The analysis of continuous variables following a normal distribution was conducted using mean ($\bar{X}$) ± standard deviation and independent sample *t*-tests. For continuous variables not conforming to a normal distribution, the study utilized median (M) and interquartile range (IQR) alongside Mann-Whitney U tests for comparison analysis. Categorical variables were represented using percentages (*n*, %) and analyzed using chi-square tests (*χ*^2^), continuity correction, or Fisher’s exact test.

Multiple comparisons of continuous variables conforming to a normal distribution and variance homogeneity were performed using one-way ANOVA with Bonferroni correction for adjusting *P*-values. Continuous variables with non-normally distributed and irregularity of variance were analyzed using Kruskal-Wallis one-way ANOVA with all pairwise comparison for adjusting *P*-values. PCA was plotted using https://www.bioinformatics.com.cn (last accessed on 6 March 2024), while the heatmap was performed by Hiplot Pro (https://hiplot.com.cn). The multiple linear regression model of log2-transformed cytokines on SFTS and HFRS was performed using the *lm*() function in R Statistical Software, and *P*-values were adjusted using the Storey false discovery rate in the *qvalue* package, defining group as the independent variable, gender and age as covariates, analyzing whether the effect of group on cytokines was influenced by age and gender, and initially screening for group-related cytokines with *q*-values <0.05 (22). Scatter plot of relative concentrations of cytokines selected from multiple linear regression and fold changes of significantly different cytokines was visualized using the *ggplot2* package in R Statistical Software. Subsequently, a random forest model of log2-transformed cytokines was constructed using the *Random Forest* package in R Statistical Software for final selection based on the results of the preliminary screening, which randomly divided the data into training and validation sets in an 8:2 ratio and determined the best seed number (500) and mtry value (5). Fivefold cross-validation was performed to draw the error rate curve, and the top 10 cytokines were selected using the mean decrease accuracy method for drawing receiver operating characteristic (ROC) curves in the validation set. SPSS 25.0 software and R Studio 4.2.2 were used for all statistical analyses, while GraphPad Prism 9.0 and R Studio 4.2.2 were utilized for graphical representations. A two-sided *P*-value less than 0.05 was considered statistically significant.

## RESULTS

### Baseline and clinical characteristics

**Fig 1:**
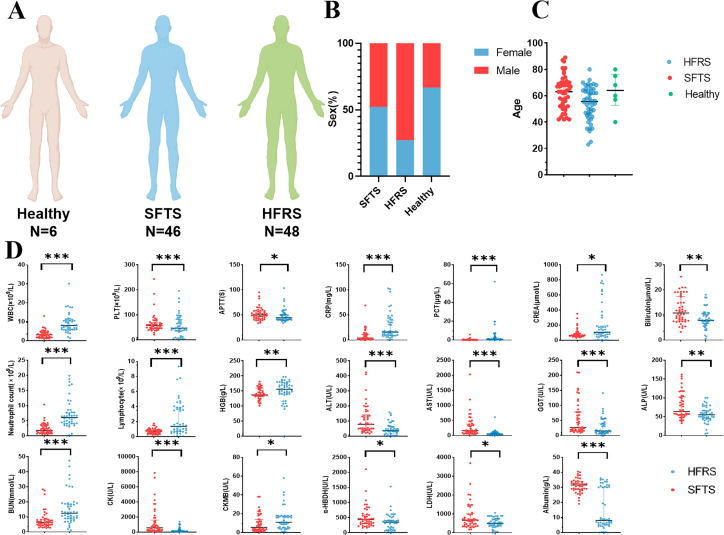
Baseline characteristics of the study cohort. (**A**) Number of individuals in the healthy control, SFTS, and HFRS groups. (**B and C**) Distribution of gender and age in the healthy control, SFTS, and HFRS groups. (**D**) Comparison of laboratory results between SFTS and HFRS patients at baseline (within 12 hours of admission). Abbreviations: WBC, white blood cell; HGB, hemoglobin; PLT, platelet; CRP, C-reactive protein; PCT, procalcitonin; BUN, blood urea nitrogen; CREA, creatinine; CK, creatine phosphokinase; ALT, alanine aminotransaminase; AST, aspartate aminotransferase; CK-MB, creatine kinase Isoenzyme-MB; ALP, alkaline phosphatase; CK, creatine kinase; LDH, lactate dehydrogenase; α-HBDH, α-hydroxybutyrate dehydrogenase; GGT, γ-glutamyl transferase. *, *P*＜0.05； **, *P*＜0.01；***, *P*＜0.001.

A total of 100 cases, including 46 cases of SFTS patients, 48 cases of HFRS patients, and 6 healthy controls, were studied (Fig. 1A). The baseline characteristics and laboratory parameters of the study cohorts are presented in Table 1 and Table S1. There were no differences between the training and validation sets regarding baseline and clinical characteristics (*P*＞0.05).

**TABLE 1:** Baseline demographics and clinical characteristics of SFTS and HFRS patients and randomized training and validation^*c*^^,^^*d*^

Characteristics	Healthy (*n* = 6)	SFTS (*n* = 46)	HFRS (*n* = 48)	*P*-value^*a*^	Training set (*n* = 75)	Validation set (*n* = 19)	*P*-value^*b*^
Age, years	64 (52.8–76.2)	62.6 ± 12.7	54.3 ± 12.9	0.002	58.3 ± 13.1	57.7 ± 15.1	0.867
≤45	1 (16.7)	5 (10.9)	11 (22.9)	0.129	13 (17.3)	4 (21.1)	0.798
45–60	2 (33.3)	14 (30.4)	18 (37.5)		24 (32.0)	7 (36.8)	
>60	3 (50.0)	27 (58.7)	19 (39.6)		38 (50.7)	8 (42.1)	
Sex, male	2 (33.3)	22 (47.8)	35 (72.9)	0.013	49 (65.3)	8 (42.1)	0.064
Highest temperature,°C		38.7 ± 0.6	38.2 (36.9–38.9)	0.001	38.6 (38.0–39.0)	38.3 ± 1.0	0.450
Clinical manifestation							
Arthralgia		2 (4.3)	8 (16.7)	0.109	7 (9.3)	3 (15.8)	0.690
Nausea		14 (30.4)	25 (52.1)	0.033	31 (41.3)	8 (42.1)	0.951
Vomiting		9 (19.6)	18 (37.5)	0.055	22 (29.3)	5 (26.3)	0.795
Abdominal pain		2 (4.3)	9 (18.8)	0.030	9 (12.0)	2 (10.5)	1.000
Diarrhea		11 (23.9)	0	<0.001	8 (10.7)	3 (15.8)	0.825
Melena		0	1 (2.1)	1.000	1 (1.3)	0	1.000
Hyperemia of conjunctiva		2 (4.3)	26 (54.2)	<0.001	24 (32.0)	4 (21.1)	0.351
Lower extremity edema		0	3 (6.3)	0.256	2 (2.7)	1 (5.3)	0.496
Percussion tenderness on kidney region		1 (2.2)	16 (33.3)	<0.001	12 (16.0)	5 (26.3)	0.478
Oliguria		0	23 (47.9)	<0.001	18 (24.0)	5 (26.3)	1.000
Conscious disturbance		1 (2.2)	1 (2.1)	1.000	2 (2.7)	0	1.000
Mucocutaneous hemorrhage		2 (4.3)	4 (8.3)	0.713	5 (6.7)	1 (5.3)	1.000
Enlarged lymph nodes		15 (32.6)	1 (2.1)	<0.001	11 (14.7)	5 (26.3)	0.387
Hepatosplenomegaly		0	1 (2.1)	1.000	1 (1.3)	0	1.000
Abdominal tenderness		2 (4.3)	9 (18.8)	0.030	10 (13.3)	1 (5.3)	0.563
Neurological signs		2 (4.3)	1 (2.1)	0.970	3 (4.0)	0	1.000

^*a*^ *P*-values comparing the group of SFTS and HFRS.

^*b*^ *P*-values comparing the group of training cohorts and validation cohorts.

^*c*^ Continuous variable data are presented as mean (SD) and median (IQR). Classified variables are represented with frequency.

^*d*^ SFTS: severe fever with thrombocytopenia syndrome, HFRS: hemorrhagic fever with renal syndrome.

Significant distinctions were noted between the two diseases in terms of age, gender ratio, and highest body temperature (Table 1; Fig. 1B and C). The proportion of individuals aged over 60 years was the highest in both diseases, but the percentage of SFTS patients over 60 years old (58.7%) was higher than that of HFRS patients (39.6%) (*P* = 0.129). The age of SFTS patients (62.6 ± 12.7) exceeded that of HFRS patients (54.3 ± 12.9) (*P* = 0.002) (Fig. 1C), and the proportion of males among HFRS patients (72.9%) was higher than that among SFTS patients (47.8%) (*P* = 0.013) (Fig. 1B). The highest body temperature of SFTS patients (38.7°C ± 0.6°C) exceeded that of HFRS patients 38.2 (36.9°C–38.9°C) (*P* = 0.001). Nausea, abdominal pain, conjunctival congestion, oliguria, percussion pain in the renal region, and abdominal tenderness were more frequent in HFRS patients than in SFTS patients, while diarrhea and enlarged lymph nodes were more common in SFTS patients than in HFRS patients (*P* < 0.05). Platelet counts, TBIL, ALT, AST, GGT, ALP, CK, α-HBDH, LDH, albumin, and coagulation time were higher in SFTS patients than in HFRS patients, whereas WBC, neutrophil, and lymphocyte counts, HGB, CRP, BUN, CREA, and CK-MB, were higher in HFRS patients than in SFTS patients (*P* < 0.05) (Table S1; Fig. 1D).

### Cytokine elevations in SFTS and HFRS compared with healthy controls

**Fig 2:**
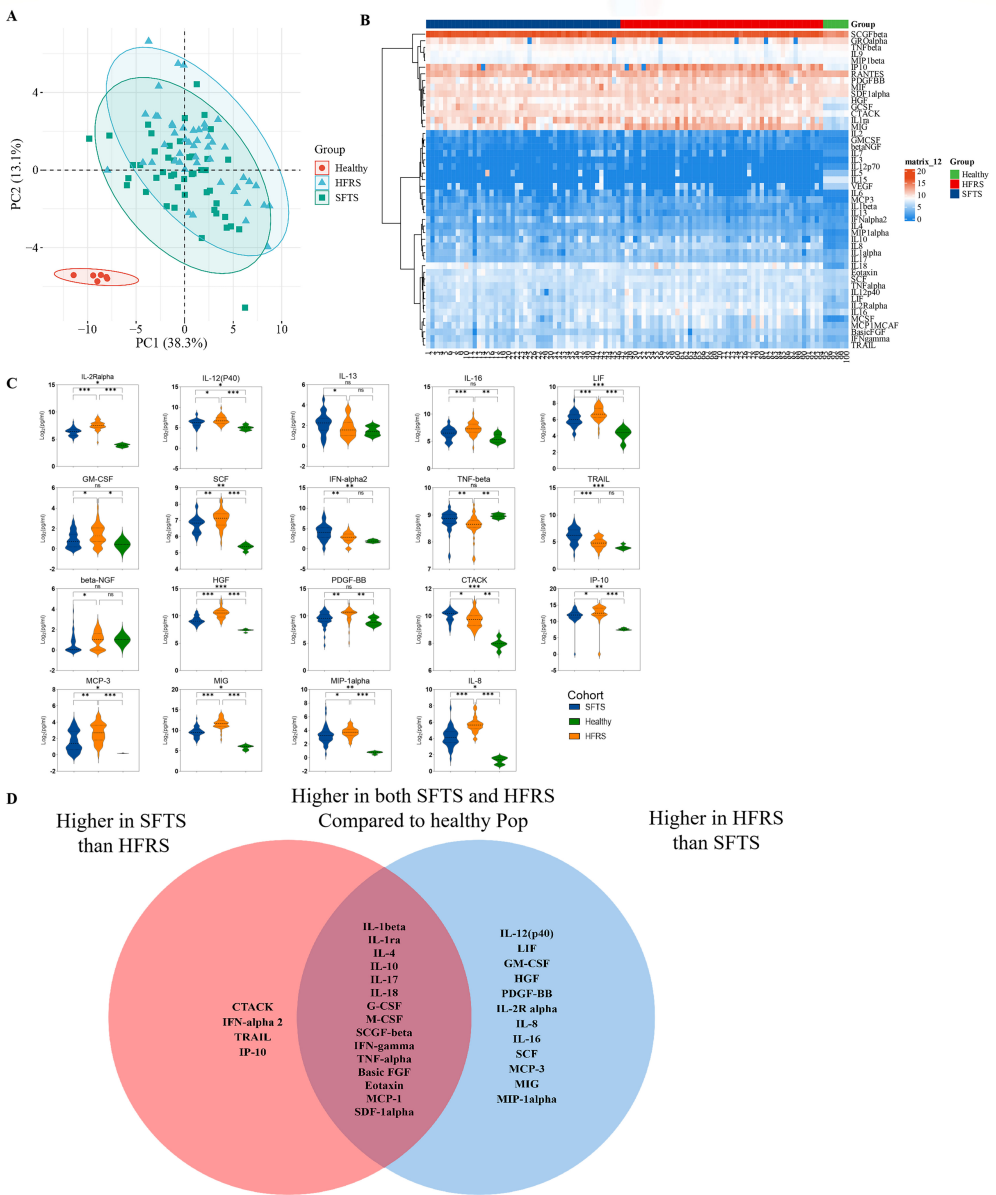
Disease-specific cytokine characteristics in SFTS and HFRS. (**A**) PCA based on the levels of all proteins. (**B**) Heatmap displaying all proteins (*x*-axis) per group with hierarchical clustering (distance: Euclidean). The color-coding boxes indicate the level of expression of the cytokine; the higher the expression, the darker the color (red is upregulated, and blue is downregulated). (**C**) Cytokines with noteworthy distinctions among healthy controls, SFTS, and HFRS. (**D**) Venn diagram illustrating cytokines that are elevated in the SFTS cohort compared to HFRS and healthy controls (left), in HFRS compared to SFTS and healthy controls (right), and in both SFTS and HFRS compared to healthy controls without significant variation between SFTS and HFRS (overlap center).

Principal component analysis (PCA) of the three cohorts revealed clustering of the SFTS and HFRS together in the principal component space, suggesting a similar level of overall inflammation between the two subgroups and significant differences compared to healthy individuals (Fig. 2A). Similarly, the heatmap revealed differences in cytokine expression among the three groups (Fig. 2B).

Among the 48 cytokines tested, 30 were found to be higher in SFTS and/or HFRS compared to the healthy control group (Fig. 2C; Fig. S1). Specifically, a total of 15 cytokines, including IL-β, IL-ra, and IL-4, exhibited increased levels in both SFTS and HFRS compared to healthy controls when SFTS and HFRS without significance (Fig. 2D). In contrast, IL-3, IL-5, IL-7, IL-12 (P70), IL-15, and VEGF were significantly decreased in SFTS and HFRS compared with HCs. IFNalpha2 and TRAIL were elevated in SFTS exclusively, not HFRS, compared with HCs. Conversely, IL-1alpha, IL-16, GM-CSF, and PDGFBB were significantly elevated solely in the HFRS cohort, with no difference between SFTS and HCs (Fig. 2C; Fig. S1; Table S2).

### Differences in cytokines between SFTS and HFRS

There were 19 different cytokines identified between SFTS and HFRS, including IL-2Ralpha, IL-8, IL-12 (P40), IL-13, IL-16, LIF, GM-CSF, SCF, β-NGF, IP-10, HGF, IFN-alpha2, CTACK, MIG, TNF-β, TRAIL, PDGFBB, MCP-3, and MIP-1alpha (Fig. 2C; Fig. S1; Table S2). Among HFRS patients, 12 cytokines (such as IL-2Ralpha, IL-8, PDGFBB, HGF, MIG, and so on) exhibited higher levels compared to SFTS and HCs. Conversely, SFTS patients demonstrated elevated levels of four cytokines (CTACK, TRAIL, IFN-alpha2, and IP-10) compared to HFRS and HCs (Fig. 2D).

**Fig 3:**
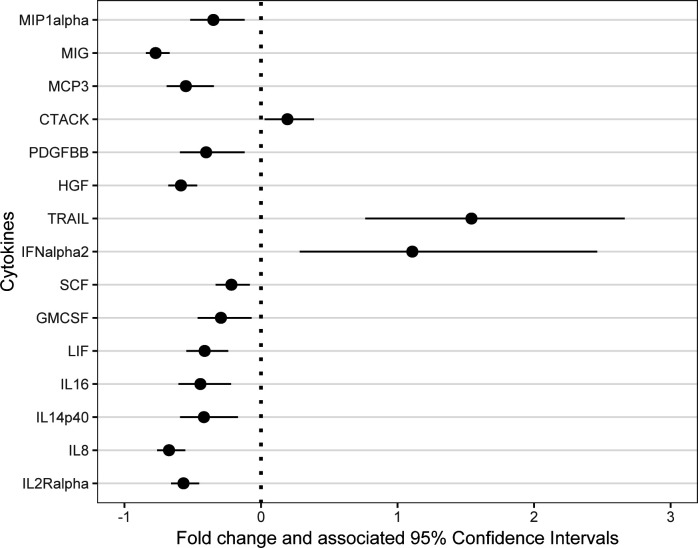
Fold changes and associated 95% confidence intervals of significantly different cytokines between SFTS and HFRS patients after adjustment for gender and age. For each cytokine, the dot represented the fold change calculated from exponentiated model coefficients, and the line represented the associated 95% CI.

**Fig 4:**
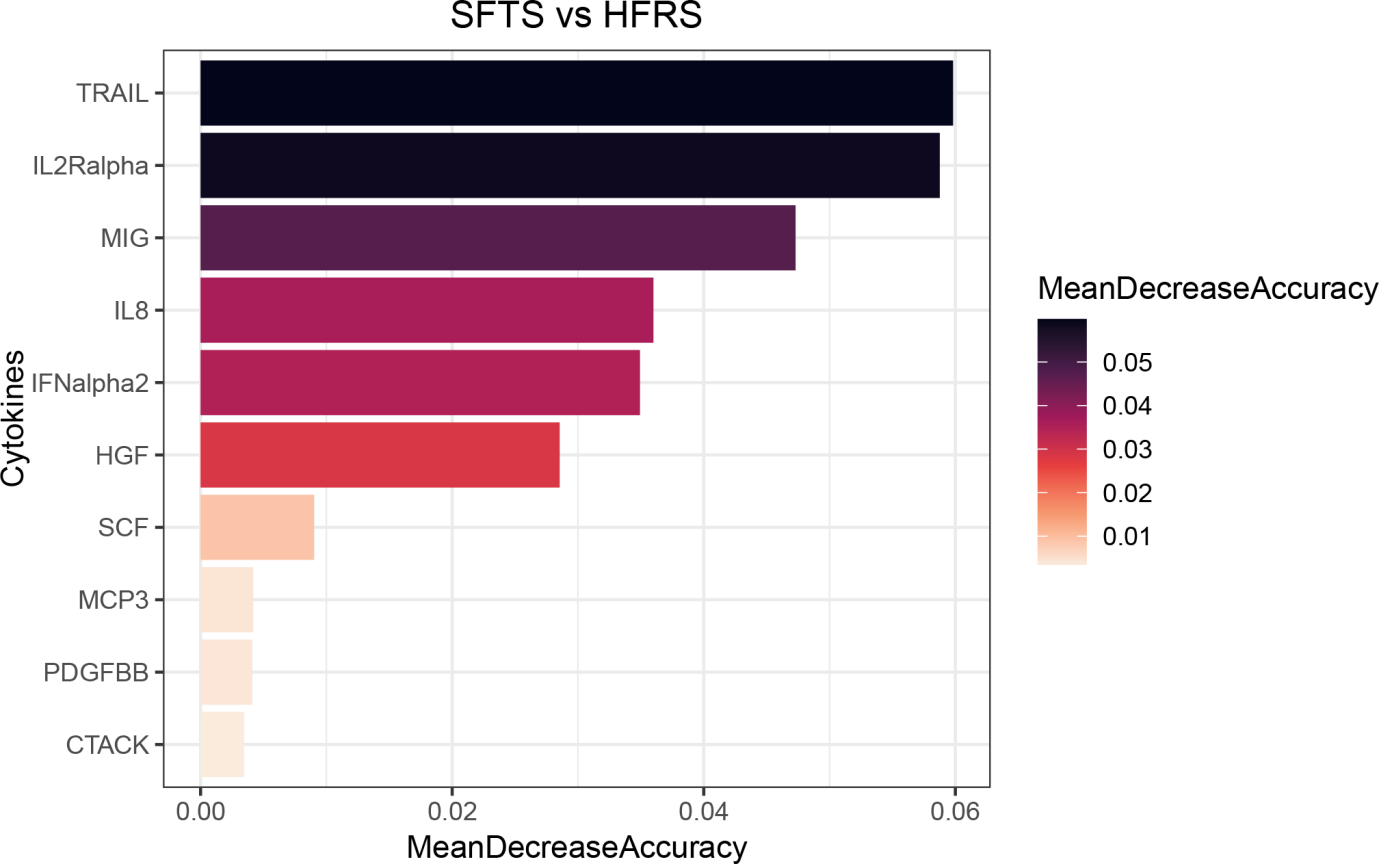
Random forest analysis revealed the most important variables in distinguishing SFTS and HFRS based on mean decrease accuracy. The random forest model for predicting SFTS versus HFRS provided feature importance, with color indicating the log2 transformation of the analyte signal between SFTS and HFRS.

**Fig 5:**
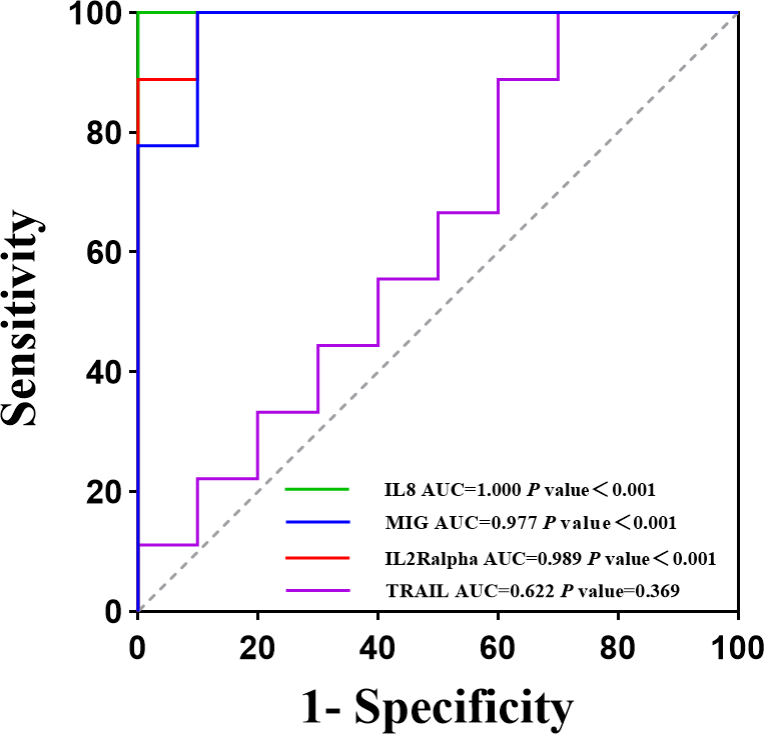
The cytokine performance in distinguishing between SFTS and HFRS diseases was evaluated. The receiver operating characteristic curve exhibited the performance of IL2Ralpha (AUC = 0.989, *P*＜0.001), MIG (AUC = 0.977, *P*＜0.001), and IL-8 (AUC = 1.000, *P*＜0.001) in differentiating HFRS from SFTS, TRAIL (AUC = 0.622, *P* = 0.369) in differentiating SFTS from HFRS in the validation set.

To further differentiate the cytokines between these two diseases, a multiple linear regression model was first developed to screen for 15 cytokines, which were IL-2Ralpha, IL-8, IL-12 (p40), IL-16, LIF, GM-CSF, SCF, IFN-alpha2, TRAIL, HGF, PDGFBB, CTACK, MCP-3, MIG, and MIP-1alpha (*q*-value <0.05) (Fig. 3; Table S3; Fig. S2). Then, these 15 cytokines were included in the random forest model for final screening, identifying the top 10 cytokines as TRAIL, IL-2Ralpha, MIG, IL-8, IFNalpha2, HGF, SCF, MCP-3, PDGFBB, and CTACK (Fig. 4). Finally, ROC curves for the 10 cytokines in the validation set were generated, indicating that only TRAIL (AUC = 0.622, *P* = 0.369) and CTACK (AUC = 0.689, *P* = 0.165) could differentiate SFTS, whereas MIG (AUC = 0.978, *P* < 0.001) and IL-2Ralpha (AUC = 0.988, *P* < 0.001) were the most effective in predicting HFRS (Fig. 5). For detailed information on the ROC curves, AUC values, and cut-off values for the top 10 cytokines, refer to Table S4 and Fig. S3.

## DISCUSSION

Both viruses belong to the order Bunyaviridae, and the mutational evolution of SFTSV and Hantaan virus remains incompletely comprehended (23, 24). Consequently, co-infection may occur as the viruses evolve. An in-depth understanding of the immune mechanisms underlying these two diseases is of significance for mitigating potential public health impacts. Consequently, our study describes the immune profiles of the two diseases, screens for characteristic immune factors, and establishes a preliminary theoretical basis for the identification of the two diseases and the exploration of potential therapeutic targets.

Previous studies of the two diseases have primarily focused on comparisons of baseline characteristics, clinical manifestations, and laboratory tests. For instance, Lu et al. discovered discrepancies in age and gender between the two diseases upon admission (12). Established studies have indicated that SFTS patients were more likely to demonstrate symptoms of diarrhea and lymph node enlargement, and higher platelet count and prothrombin time than HFRS patients, while HFRS patients were more prone to symptoms like nausea, abdominal pain, conjunctival congestion, oliguria, percussion tenderness over kidney region, abdominal pain, and lower WBCs (1, 3, 25, 26). Our findings align with these observations and validate the reliability of our research. Additionally, previous researches only depicted the immunological factor profile of one disease. Our study stands out as the first to comparatively describe the immunological factors of both diseases. We found that TRAIL and CTACK were more closely associated with SFTS, whereas IL-2Ralpha, MIG, IL-8, IFNalpha2, HGF, SCF, MCP-3, and PDGFBB were more specific with HFRS.

### TRAIL and CTACK identify SFTS better

Studies have identified splenic and hepatic macrophages as the main target cells for SFTSV infection (27). SFTSV can infect macrophages *in vivo* and cause elevated both pro-inflammatory cytokines (such as IL-6 and IFN-γ) and anti-inflammatory cytokines (such as IL-10) in SFTSV-infected patients, and SFTSV infection of macrophages contributed to macrophage differentiation into the M2 phenotype (28). M2 macrophages promote viral shedding and dissemination by targeting STAT1 (8). M1 macrophages are characterized by the secretion of proinflammatory cytokines and high phagocytosis (29), while M2 macrophages mainly produce anti-inflammatory cytokines and immunomodulatory molecules (30). Previous studies have shown that soluble TRAIL induces proinflammatory cytokine expression in human monocyte-derived M1 macrophages and mouse peritoneal M1 macrophages (31). TRAIL, also known as Apo-2 ligand or TNF receptor superfamily member 10 (TNFRSF10), binds and activates death receptors 4 and 5 (DR4 and DR5, also known as TNFRSF10A and TNFRSF10B), initiating the extrinsic pathway of apoptosis (32). TRAIL induces apoptosis in alveoli and lung macrophages of mice infected with *Streptococcus pneumoniae,* aiding in the clearance of bacteria from the respiratory tract and limiting inflammation and tissue damage (33). In addition, SFTSV infection leads to platelet activation, which, in turn, causes neutrophil activation through platelet-neutrophil agglutination, resulting in a unique mode of cell death in neutrophils (i.e., release of mitochondrial NETs that restrict pathogen dispersal) (34). TRAIL also plays a role in clearing neutrophils during activation, stress, or senescence *in vivo* (35). In conclusion, TRAIL may be involved in stimulating immune cells and triggering cytokine release during SFTSV infection.

SFTS is a tick-borne disease characterized by a localized inflammatory reaction of varying degrees in the skin occurring 24–48 hours after a tick bite, ranging from mild cases with localized erythema and a central bruise or petechiae to severe cases with evident edematous erythema, papules, or blisters around the bruise area, which may later develop into firm nodule. It is possible that this skin inflammation is mediated by CTACK, a protein that is expressed exclusively in the skin and selectively attracts a specific subset of skin memory T cells by interacting with a certain CCR10 receptor (36). The “CTACK/CCR10” interactions directly mediate the recruitment of T cells to the inflamed skin and are upregulated in the damaged skin (37). Furthermore, CTACK-induced recruitment of tumorigenic T cells to the skin is a critical pathogenic phase of mycosis fungoides, a condition characterized by the progressive clonal expansion of CD3+/CD4+/CD8− atypical lymphoid cells with skin-homing properties, resulting in the formation of skin plaques (38). Exposure to acute stress causes an increased level of CTACK gene expression. Furthermore, short-term stress exposure leads to a shift in cytokine homeostasis toward conditions that support the development and maintenance of cellular immunity (39). Therefore, tick bites, being a source of stress, can activate cellular immunity via CTACK pathway.

### IL-2Ralpha, MIG, IL-8, IFNalpha2, HGF, SCF, MCP-3, and PDGFBB identify HFRS better

CD8+ T cells are critical for eliminating virus-infected cells and viruses in patients with HFRS; both excessive and weak T cell responses can lead to severe disease (40). Among the most vital signaling pathways altered early by hantaviruses are cytokine-cytokine receptor interactions, chemokine signaling, STAT signaling, MAPK signaling, and neurotrophic factor signaling, and there is a significant increase in antigen processing and presentation pathways, as well as the cytotoxicity of NK cells (41). Previous investigation has suggested that the rate of revitalization and alteration of memory B cells is significantly accelerated in moderate cases of HFRS patients compared to severe cases; thus, the rapid expansion of HTNV-specific B cells in the early stages of infection is closely connected to the disease outcome (42). It is likely that IL-2Ralpha, MIG, IFNalpha2, SCF, and PDGFBB are involved in the activation of the immune pathways and immune cells mentioned above.

During thymic T-cell development, IL-2Ralpha (CD25) is expressed in early double-negative thymocytes and is highly expressive in the majority of regulatory T cells (Tregs), and IL-2 promotes immune tolerance in Tregs through the JAK-STAT5 pathway (43, 44, 45). CXCR3 and its ligand, MIG (CXCL9), are part of the chemokine system and play a role in the migration of effector T cells, NK cells, and B cell subsets in the inflammatory response of lymph nodes *in vivo* (46). Once bound to the receptor, MIG induces a conformational change in CXCR3, activates coupled G proteins, and initiates signaling pathways including the PI3K/AKT pathway (47). Type I interferon, consisting of IFN-β and at least 13 different IFN-as in humans, and type II IFN (IFN-γ), have antiviral effects against a variety of viruses, including hantaviruses (48). Precedent findings suggest that the absence of systemically elevated IFN-a levels in patients indicates that the IFN response is suppressed, leading to a failure to restrict HFRS infection. Suppression of the innate immune response, particularly the production and/or function of antiviral interferons, facilitates viral dissemination *in vivo* (49). However, activation of the intracellular antiviral state of IFN-a is associated with the phosphorylation of STATs (50). Upon binding of IFN-a to the common IFN type I receptor, the Janus kinases JAK-1 and TYK-2 phosphorylate STAT1 and STAT2, and then the phosphorylated STAT1/STAT2 heterodimer recruits IFN regulatory factors to form the IFN-stimulated gene factor 3 complex (49). SCF is a major growth factor for mast cells and a proinflammatory cytokine secreted by fibroblasts, stromal cells, and endothelial cells (ECs) (51). The binding of SCF to C-kit initiates receptor dimerization and autophosphorylation of tyrosine residues in the cytoplasmic structural domain of the receptor, activating various intracellular signaling cascades such as Janus kinase/signal transducer activator of transcription (JAK/STAT) and mitogen-activated protein kinase/EC signal-regulated kinase (MAPK/ERK) (52). Besides, SCF binds to B-Cell Kit (CD117) receptor and secretes cytokines like IL-7 to promote the growth of B cells (51). PDGF-BB has chemotactic effects on inflammatory cells, such as neutrophils and macrophages (53), and it can activate a variety of signaling pathways, including PI3K/AKT, MAPK, JAK/STAT, and PLC-γ, which are related to anti-apoptotic and proliferative effects, tissue development, induction of antioxidant proteins, and mitochondrial protection (54).

Besides, NETs have been previously detected in renal biopsies of hantavirus-infected patients and in mouse models, suggesting that NETs induce renal injury with signs of extracapillary glomerulonephritis and/or tubulointerstitial nephritis (55). NETs are large extracellular reticular structures composed of neutrophil cytoplasm and granule proteins that trap, neutralize, and kill bacteria, fungi, viruses, and parasites (56). Following hantavirus infection, the expression of HLA (human leukocyte antigen) class I molecules is increased on endothelial cells, and this allows hantavirus-infected EC to interact with a wide range of immune effector cells, such as HLA class I-restricted CD8+ T cells and neutrophils. Neutrophils are known to contribute to hantavirus-induced immunopathogenesis, producing NETosis upon interaction with activated EC. Additionally, neutrophils express β2 integrins, and hantavirus can activate neutrophils through β2 integrin signaling or platelet-neutrophil interactions via β2 integrins, leading to NETosis (57). IL-8 is an essential neutrophil chemokine, and neutrophil/NETs-mediated EC injury depends on IL-8 production by activated ECs (57). Moreover, NETs formation involves IL-8 and HGF, which stimulate neutrophils to release IL-8, both of which together activate CXCR1 and CXCR2 receptors on neutrophils to induce NET formation (51).

The principal target organ of hantavirus is renal injury. Prior studies have revealed that HGF is a potent anti-inflammatory factor, whose overexpression significantly protects the kidneys from ischemia-induced acute renal failure (58). However, during acute kidney injury (AKI), renal B cells become an important source of MCP-3, promoting neutrophil and monocyte recruitment to exacerbate the severity of AKI (59). Furthermore, renal mesangial cell growth is substantially driven by PDGF, and the expression of PDGF ligand and receptors is lower in normal adult kidneys but increases during the advance of renal development and renal fibrogenesis (60). Historically, Cizman et al. reported chronic renal insufficiency and renal interstitial fibrosis in patients who had recovered 2 years after the HFRS outbreak in Yugoslavia (61). Therefore, the overexpression of PDGF-BB released from platelet alpha granules may be associated with organ fibrosis in HFRS patients.

In summary, the majority of the top 10 cytokines screened in our study identified HFRS, and only two correlated with SFTS. The top 4 cytokines were TRAIL, IL-2Ralpha, MIG, and IL-8, of which TRAIL had a *P*-value >0.05 in the validation set, which may be related to the small sample size of the validation set. There are several limitations to our study: (i) Given the incomplete coverage of disease extent in the study region, our research only included patients with the common types of these two diseases. (ii) Our study is a single-center study with limited sample volume and lacks multicenter validation. (iii) Cytokine levels were only measured on the day of admission, and serial sampling was not performed for disease progression analysis. (iv) There was no further experimental validation to confirm that the selected cytokines are specific to these two diseases. These limitations will be addressed in future studies to improve the comprehensiveness and reliability of our findings.

### Conclusion

Our research was the first to characterize the immune factor profiles of SFTS and HFRS. Elevated acute-phase plasma levels of IL2Ralpha, MIG, and IL-8 are more common in patients with HFRS, while elevated levels of TRAIL are more common in patients with SFTS. Cytokine measurement can assist in formulating clinical therapeutic strategies, such as using cytokine antagonists to prevent disease progression. Further research is needed to better elucidate the immune responses in both SFTS and HFRS to inform diagnostic strategies and potential therapies.

## Data Availability

All relevant data are within the article and its supplemental material.

## SUPPLEMENTAL MATERIAL

Supplemental figure legends jvi.00786-24-s0001.docx Legends for Fig. S1 to S3.

Fig. S1 jvi.00786-24-s0002.tif Cytokines with significant differences among healthy controls, SFTS, and HFRS.

Fig. S2 jvi.00786-24-s0003.tif Relative (log2-transformed) concentration of significantly different cytokines between SFTS and HFRS patients after adjustment for gender and age.

Fig. S3 jvi.00786-24-s0004.tif Receiver operator characteristics (ROC) curve showing the performance of the other six cytokines that differentiate HFRS disease and SFTS in the validation set.

Graphical abstract legend jvi.00786-24-s0005.docx Legend for graphical abstract.

Graphical abstract jvi.00786-24-s0006.png TRAIL, IL-2Ralpha, MIG, and IL-8 were identified as the top four cytokines that effectively differentiate between SFTS and HFRS.

Table S1 jvi.00786-24-s0007.docx Laboratory parameters of SFTS and HFRS.

Table S2 jvi.00786-24-s0008.docx Comparison of Log2-converted cytokines among healthy, HFRS, and SFTS groups.

Table S3 jvi.00786-24-s0009.docx Statistically significant cytokines as determined by multiple linear regression analysis.

Table S4 jvi.00786-24-s0010.docx Predictive value of the top 10 cytokines extracted from the random forest model in predicting SFTS and HFRS.

ASM does not own the copyrights to Supplemental Material that may be linked to, or accessed through, an article. The authors have granted ASM a non-exclusive, world-wide license to publish the Supplemental Material files. Please contact the corresponding author directly for reuse.
