# Supplementary material for: The differences in cytokine signatures between severe fever with thrombocytopenia syndrome (SFTS) and hemorrhagic fever with renal syndrome (HFRS)
Source: J Virol. 2024 Jun 25;98(7):e00786-24. doi: 10.1128/jvi.00786-24 (PMC11265425; doi:10.1128/jvi.00786-24)
Supplement: Supplemental figure legends — Legends for Fig. S1 to S3. [file jvi.00786-24-s0001.docx]

Supplemental Figure legends:

Supplementary Figure S1. cytokines with significant differences among Healthy controls、SFTS and HFRS. Twenty-nine cytokines showed no significant statistical difference between the SFTS and HFRS groups.

Supplementary Figure S2. Relative (log2-transformed) concentration of significantly different cytokines between SFTS and HFRS patients after adjustment for gender and age. Red line indicated the average of a cytokines for a given group. Each dot represented a single individual (blue=SFTS; orange=HFRS).

Supplementary Figure S3. Receiver operator characteristics (ROC) curve showing the performance of the other six cytokines that differentiate HFRS disease and SFTS in the validation set. The CTACK had an AUC of 0.689 (P = 0.165). The MCP-3 had an AUC of 0.922 (P = 0.0019). The SCF had an AUC of 0.789 (P = 0.034). The HGF had an AUC of 0.922 (P = 0.019). The PDGFBB had an AUC of 0.833 (P = 0.014). The IFN alpha2 had an AUC of 0.556 (P = 0.683).
