# Supplementary figures and images for: The differences in cytokine signatures between severe fever with thrombocytopenia syndrome (SFTS) and hemorrhagic fever with renal syndrome (HFRS)
Source: J Virol. 2024 Jun 25;98(7):e00786-24. doi: 10.1128/jvi.00786-24 (PMC11265425; doi:10.1128/jvi.00786-24)

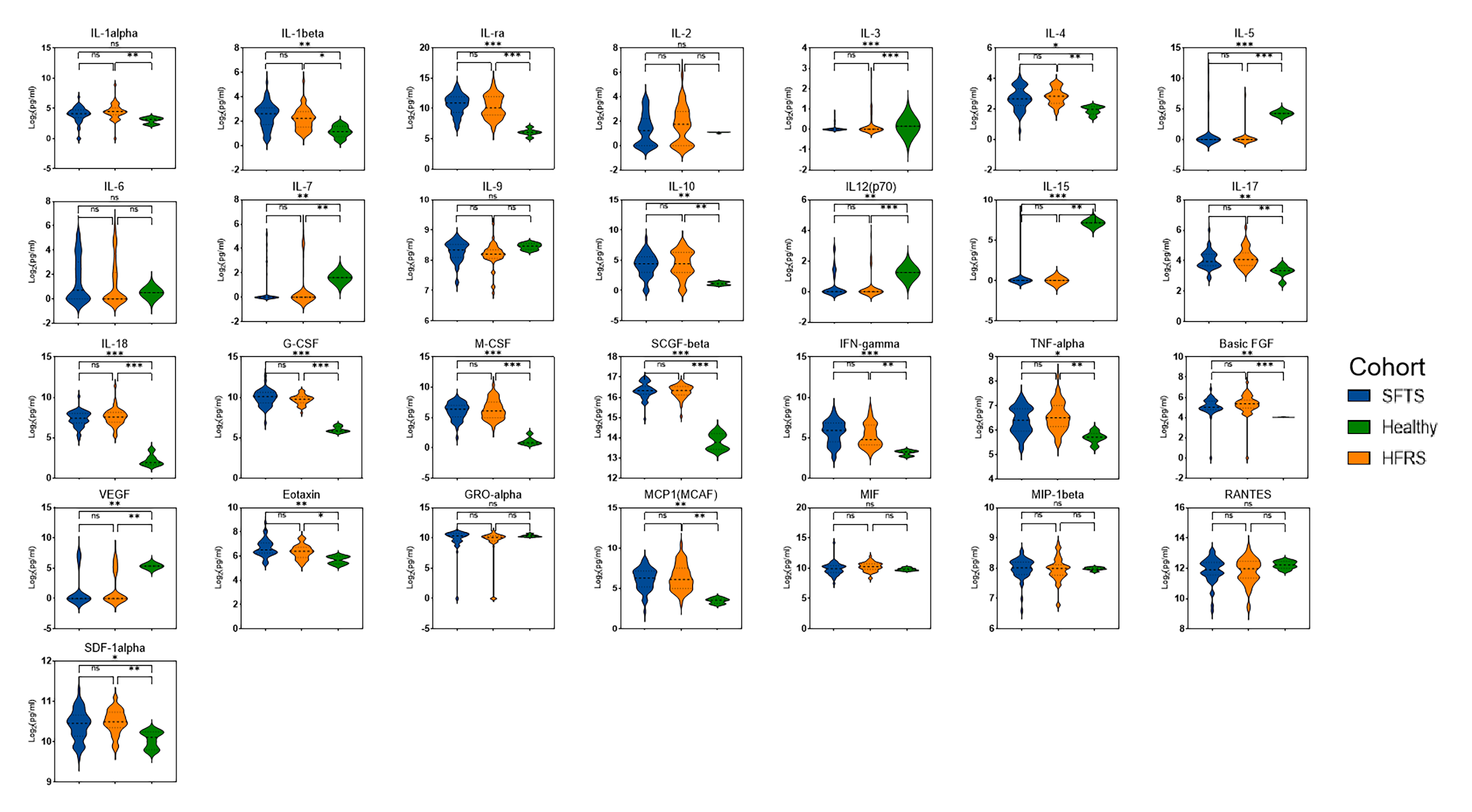

Supplement: Fig. S1 — Cytokines with significant differences among healthy controls, SFTS, and HFRS. [file jvi.00786-24-s0002.tif]

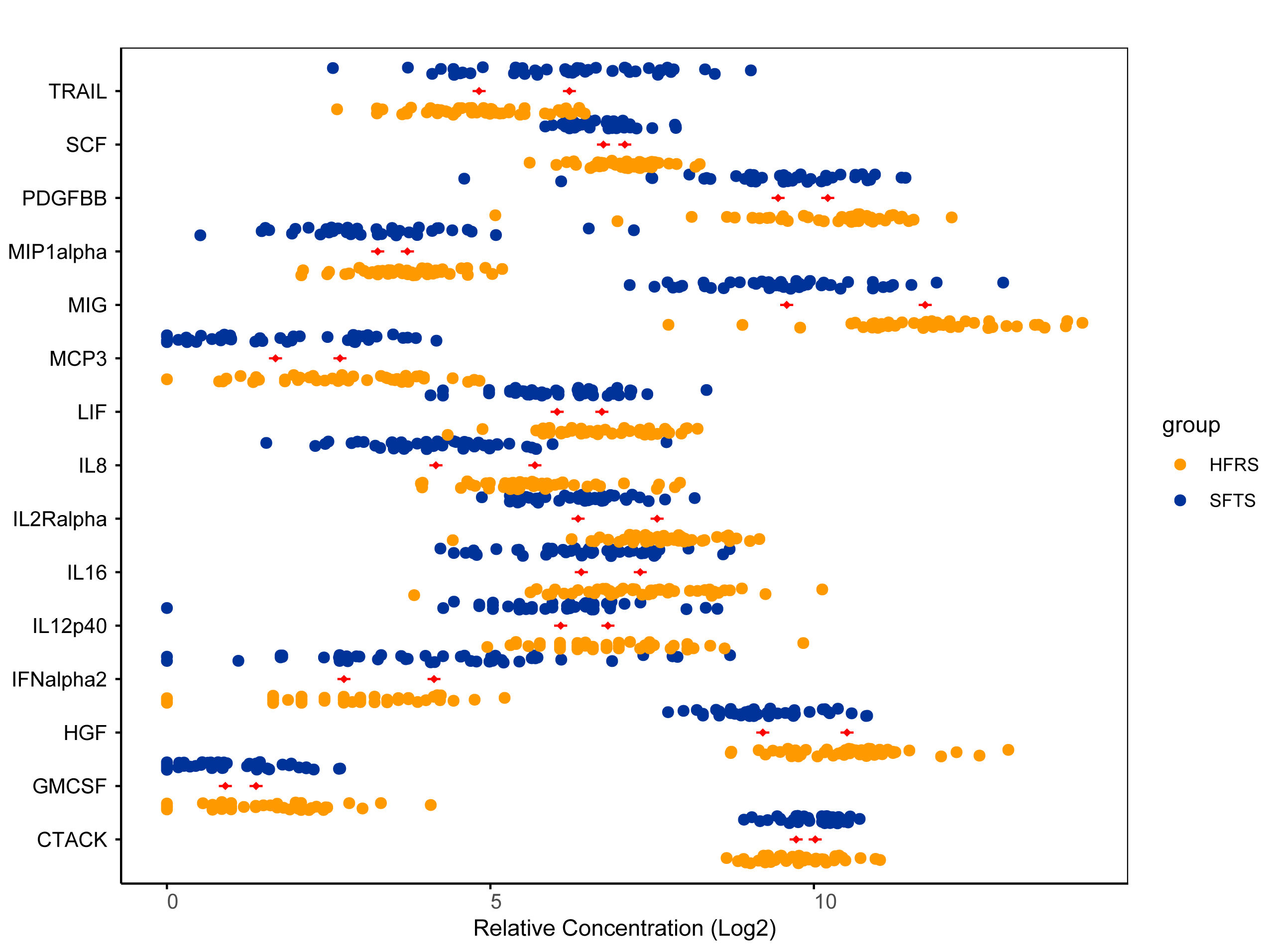

Supplement: Fig. S2 — Relative (log2-transformed) concentration of significantly different cytokines between SFTS and HFRS patients after adjustment for gender and age. [file jvi.00786-24-s0003.tif]

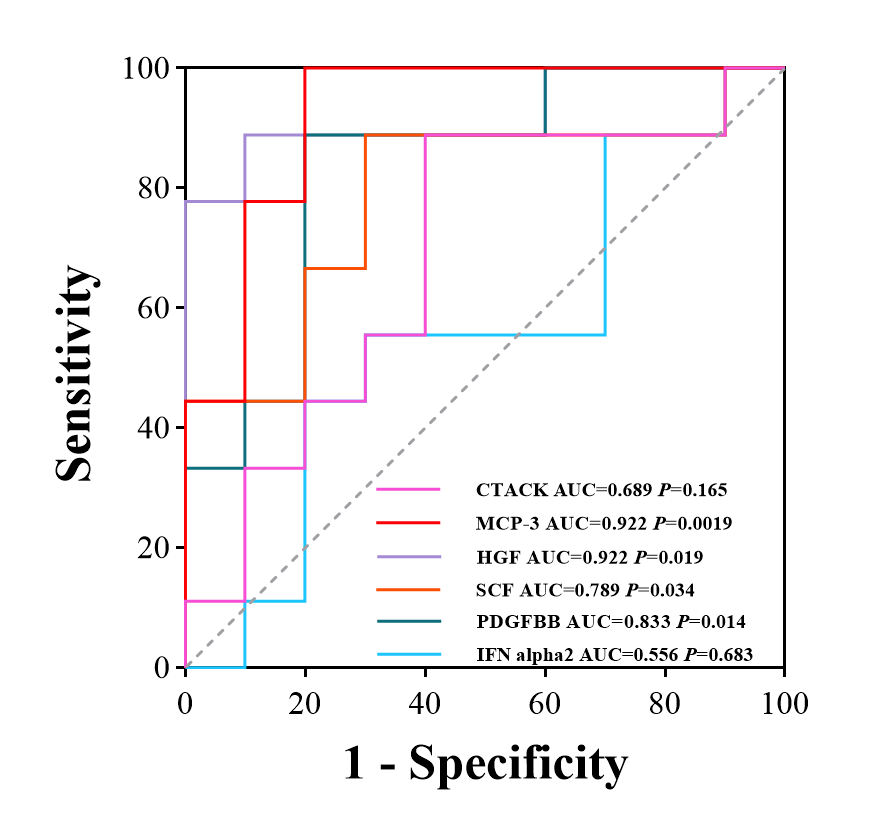

Supplement: Fig. S3 — Receiver operator characteristics (ROC) curve showing the performance of the other six cytokines that differentiate HFRS disease and SFTS in the validation set. [file jvi.00786-24-s0004.tif]

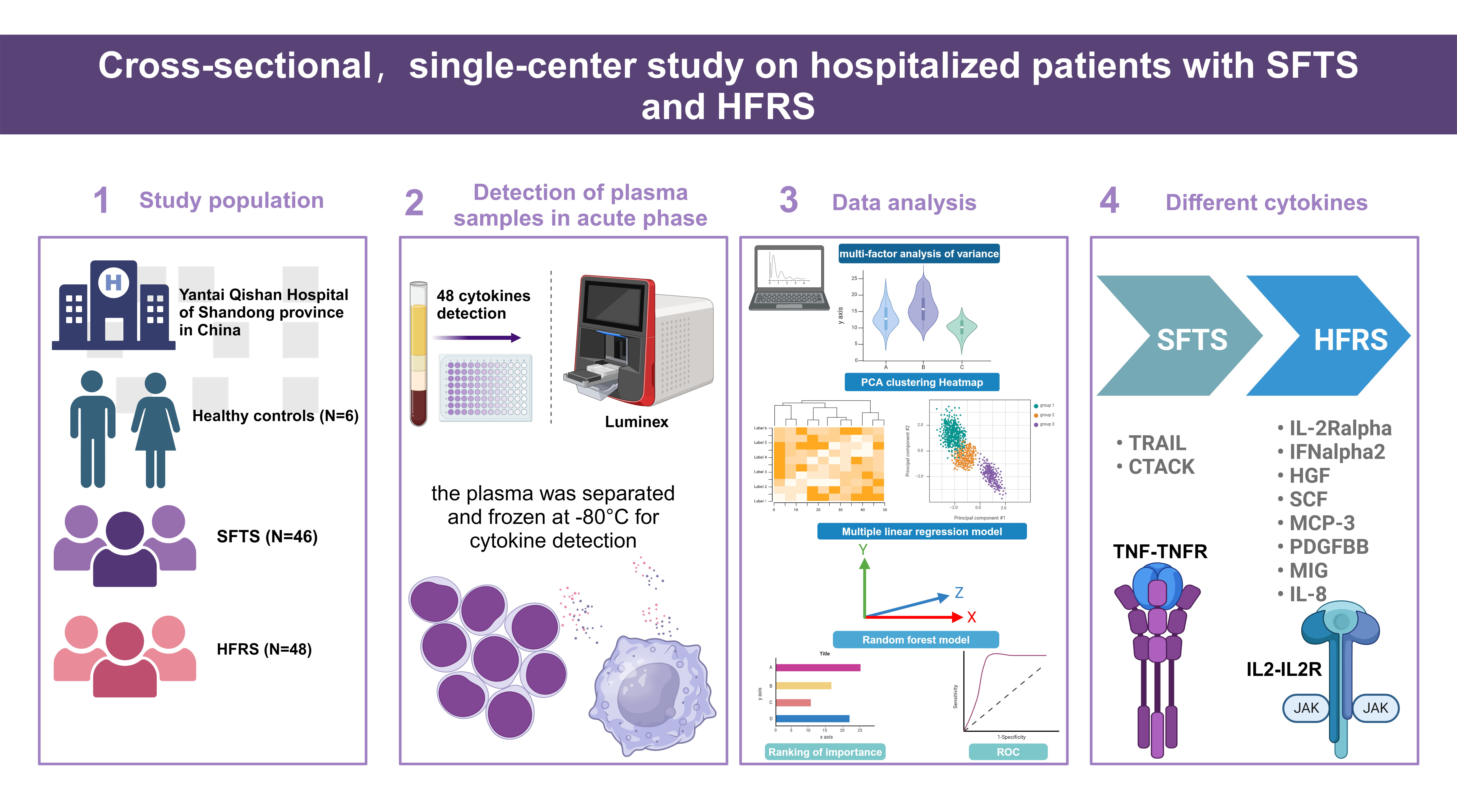

Supplement: Graphical abstract — TRAIL, IL-2Ralpha, MIG, and IL-8 were identified as the top four cytokines that effectively differentiate between SFTS and HFRS. [file jvi.00786-24-s0006.png]
