# Supplementary material for: The differences in cytokine signatures between severe fever with thrombocytopenia syndrome (SFTS) and hemorrhagic fever with renal syndrome (HFRS)
Source: J Virol. 2024 Jun 25;98(7):e00786-24. doi: 10.1128/jvi.00786-24 (PMC11265425; doi:10.1128/jvi.00786-24)
Supplement: Graphical abstract legend — Legend for graphical abstract. [file jvi.00786-24-s0005.docx]

**Graphical abstract**

Liu ZS et al. elucidated the cytokine immune profiles of two diseases, SFTS and HFRS, which share similarities in viral genes, epidemiological features, and clinical manifestations. Through multiple linear regression analysis and random forest analysis, TRAIL,IL-2Ralpha, MIG and IL-8 were identified as the top four cytokines that effectively differentiate between SFTS and HFRS.
