# Supplementary material for: The differences in cytokine signatures between severe fever with thrombocytopenia syndrome (SFTS) and hemorrhagic fever with renal syndrome (HFRS)
Source: J Virol. 2024 Jun 25;98(7):e00786-24. doi: 10.1128/jvi.00786-24 (PMC11265425; doi:10.1128/jvi.00786-24)
Supplement: Table S1 — Laboratory parameters of SFTS and HFRS. [file jvi.00786-24-s0007.docx]

| Supplementary Table S1. Laboratory parameters of SFTS and HFRS | | | | |
| --- | --- | --- | --- | --- |
| **Parameters** | **Normal range** | **SFTS**  **（n=46）** | **HFRS**  **（n=48）** | ***P* value** |
| WBC,×10^9^/L | 3.5-9.5 | 3.05(1.73-4.43) | 7.96(5.90-10.87) | ＜0.001 |
| Neutrophils, × 10^9^/L | 1.6-5.9 | 1.82(1.03-3.42) | 6.06(4.05-7.78) | ＜0.001 |
| Lymphocytes,× 10^9^/L | 1.1-3.5 | 0.73(0.48-0.91) | 1.37(0.69-3.82) | ＜0.001 |
| HGB,g/L | 115-150 | 142.04±18.92 | 156.00(140.00-176.50) | 0.007 |
| PLT,10^9^/L | 125-350 | 57.00(45.50-68.75) | 47.00(35.25-82.00) | ＜0.001 |
| CRP,mg/L | ＜ 3 | 4.07(1.94-11.47) | 15.81(9.16-39.50) | ＜0.001 |
| PCT,µg/L | ＜ 0.5 | 0.16(0.07-0.46) | 0.91(0.47-3.07) | ＜0.001 |
| TBIL,µmol/L | ＜ 26 | 10.85(7.67-17.38) | 8.51±3.73 | 0.001 |
| ALT,U/L | 7-40 | 78.90(42.3-142.38) | 36.60(17.13-61.38) | ＜0.001 |
| AST,U/L | 13-35 | 162.40(88.43-380.13) | 50.20(33.13-85.78) | ＜0.001 |
| GGT,U/L | 7-32 | 27.00(16.75-78.00) | 15.00(9.00-40.75) | ＜0.001 |
| ALP,U/L | 40-150 | 64.00(54.58-102.58) | 55.79±21.00 | 0.003 |
| BUN,mmol/L | 1.7-8.3 | 6.43(4.47-8.32) | 12.43(8.59-18.60) | ＜0.001 |
| CK,U/L | 29-168 | 573.00(246.50-1592.00) | 103.50(65.50-269.75) | ＜0.001 |
| CREA,µmol/L | ＜ 90 | 66.85(53.83-89.60) | 106.50(56.25-190.50) | 0.012 |
| CK-MB,U/L | 0-24 | 5.58(2.57-14.02) | 11.00(5.00-17.75) | 0.039 |
| α-HBDH,U/L | 72-182 | 431.64(292.86-590.81) | 351.47(94.85-411.52) | 0.012 |
| LDH,U/L | 109-245 | 667.45(408.25-1013.50) | 533.96±266.15 | 0.042 |
| Albumin,g/L | 35-53 | 31.14±4.67 | 8.09(5.20-30.40) | ＜0.001 |
| Prolonged coagulation time,s | 28-43.5 | 49.90(42.68-57.98) | 44.35(40.33-50.90) | 0.038 |

Continuous variable data were presented as mean (SD) and median (interquartile ranges, IQR). Classified variables were represented with frequency.

Abbreviations: WBC: white blood cell, HGB: hemoglobin, PLT: platelet, CRP: C-reactive protein, PCT: procalcitonin, ALT: alanine aminotransaminase, AST: aspartate aminotransferase, GGT: γ-glutamyl transferase, ALP: alkaline phosphatase, BUN: blood urea nitrogen, CREA: creatinine, CK: creatine phosphokinase, LDH: lactate dehydrogenase, CK-MB: creatine kinase Isoenzyme-MB, α-HBDH: α-hydroxybutyrate dehydrogenase, TBIL: total bilirubin.
