# Supplementary material for: The differences in cytokine signatures between severe fever with thrombocytopenia syndrome (SFTS) and hemorrhagic fever with renal syndrome (HFRS)
Source: J Virol. 2024 Jun 25;98(7):e00786-24. doi: 10.1128/jvi.00786-24 (PMC11265425; doi:10.1128/jvi.00786-24)
Supplement: Table S2 — Comparison of Log2-converted cytokines among healthy, HFRS, and SFTS groups. [file jvi.00786-24-s0008.docx]

| Supplementary Table S2. Comparison of Log2-converted cytokines among healthy, HFRS and SFTS groups | | | | | | | | | |
| --- | --- | --- | --- | --- | --- | --- | --- | --- | --- |
|  | **log2(SFTS)** | **log2 (HFRS)** |  | **SFTS vs HFRS** | | **SFTS vs Healthy** | | **HFRS vs Healthy** | |
| **Cytokines** |  |  | **log2(Healthy)** | **p-value** | **Adjusted p-value** | **p-value** | **Adjusted p-value** | **p-value** | **Adjusted p-value** |
| IL-1alpha | 4.11(3.48-4.71) | 4.46(3.96-5.12) | 2.99±0.53 | 0.077 | 0.230 | 0.017 | 0.051 | 0.001 | 0.004 |
| IL-1beta | 2.50±1.04 | 2.24(1.54-2.78) | 1.16±0.48 | 0.092 | 0.277 | 0.001 | 0.003 | 0.012 | 0.036 |
| IL-1ra | 10.70±1.67 | 10.29±1.90 | 6.08±0.59 | 0.294 | 0.883 | ＜0.001 | ＜0.001 | ＜0.001 | 0.001 |
| IL-2 | 1.24(0-2.22) | 1.77(0-2.82) | 1.12(1.09-1.12) | NS | NS | NS | NS | NS | NS |
| IL-2R alpha | 6.35±0.69 | 7.53(7.21-8.00) | 3.87±0.28 | ＜0.001 | ＜0.001 | 0.014 | 0.041 | ＜0.001 | ＜0.001 |
| IL-3 | 0(0-0) | 0(0-0) | 0.16±0 | 0.551 | 1.000 | ＜0.001 | ＜0.001 | ＜0.001 | ＜0.001 |
| IL-4 | 2.69±0.74 | 2.85±0.54 | 1.92±0.28 | 0.279 | 0.838 | 0.004 | 0.013 | 0.001 | 0.002 |
| IL-5 | 0(0-0) | 0(0-0) | 4.26±0 | 0.502 | 1.000 | ＜0.001 | ＜0.001 | ＜0.001 | 0.001 |
| IL-6 | 0.72(0-3.03) | 0(0-2.18) | 0.51±0 | NS | NS | NS | NS | NS | NS |
| IL-7 | 0(0-0.47) | 0(0-0) | 1.62±0 | 0.307 | 0.920 | 0.003 | 0.008 | ＜0.001 | 0.001 |
| IL-8 | 4.16±1.11 | 5.66(5.22-6.05) | 1.31±0.42 | ＜0.001 | ＜0.001 | 0.013 | 0.038 | ＜0.001 | ＜0.001 |
| IL-9 | 8.34(8.10-8.53) | 8.20(8.07-8.35) | 8.46±0.11 | 0.028 | 0.083 | 0.195 | 0.585 | 0.019 | 0.057 |
| IL-10 | 4.21±1.91 | 4.33±2.19 | 1.16±0.27 | 0.727 | 1.000 | 0.001 | 0.004 | 0.001 | 0.002 |
| IL-12(p40) | 6.27(5.53-6.85) | 6.82±0.98 | 4.98±0.56 | 0.008 | 0.025 | 0.008 | 0.025 | ＜0.001 | ＜0.001 |
| IL-12(p70) | 0(0-0.92) | 0(0-0) | 1.26±0 | 0.101 | 0.303 | 0.001 | 0.002 | ＜0.001 | ＜0.001 |
| IL-13 | 2.20±1.02 | 1.57(1.03-2.32) | 1.46±0.44 | 0.014 | 0.043 | 0.063 | 0.188 | 0.484 | 1.000 |
| IL-15 | 0(0-0) | 0(0-0) | 7.14±0 | 0.663 | 1.000 | ＜0.001 | ＜0.001 | ＜0.001 | 0.001 |
| IL-16 | 6.40±1.08 | 7.32±1.11 | 5.52±0.76 | ＜0.001 | ＜0.001 | 0.061 | 0.182 | ＜0.001 | 0.001 |
| IL-17 | 4.04±0.60 | 4.07(3.68-4.52) | 3.27±0.41 | 0.307 | 0.921 | 0.003 | 0.009 | 0.001 | 0.002 |
| IL-18 | 7.34±0.98 | 7.58±1.08 | 2.21±0.72 | 0.264 | 0.791 | ＜0.001 | ＜0.001 | ＜0.001 | ＜0.001 |
| LIF | 6.03±0.87 | 6.72±0.81 | 4.23±0.77 | ＜0.001 | ＜0.001 | ＜0.001 | ＜0.001 | ＜0.001 | ＜0.001 |
| G-CSF | 9.95±1.02 | 9.73±0.60 | 6.02±0.37 | 0.154 | 0.462 | ＜0.001 | ＜0.001 | ＜0.001 | 0.001 |
| GM-CSF | 0.70(0.19-1.47) | 1.28(0.70-2.07) | 0.43±0 | 0.014 | 0.041 | 0.177 | 0.531 | 0.011 | 0.034 |
| M-CSF | 6.08±1.43 | 6.35±1.62 | 0.85(0.70-1.63) | 0.709 | 1.000 | ＜0.001 | ＜0.001 | ＜0.001 | ＜0.001 |
| SCF | 6.84(6.30-7.02) | 7.08±0.52 | 5.35±0.18 | 0.002 | 0.006 | 0.001 | 0.004 | ＜0.001 | ＜0.001 |
| SCGF-beta | 16.33(16.15-16.54) | 16.34(16.12-16.54) | 13.81±0.41 | 0.644 | 1.000 | ＜0.001 | ＜0.001 | ＜0.001 | ＜0.001 |
| IFN-alpha 2 | 4.13±1.96 | 2.80(2.08-3.69) | 1.85±0.32 | 0.001 | 0.002 | ＜0.001 | 0.001 | 0.057 | 0.172 |
| IFN-gamma | 5.70±1.46 | 4.78(4.14-6.56) | 3.14±0.33 | 0.134 | 0.401 | ＜0.001 | ＜0.001 | 0.001 | 0.002 |
| TNF-alpha | 6.40±0.55 | 6.56±0.59 | 5.70±0.23 | 0.164 | 0.491 | 0.005 | 0.016 | ＜0.001 | 0.002 |
| TNF-beta | 8.88(8.69-9.01) | 8.66(8.55-8.80) | 8.96±0.08 | 0.002 | 0.007 | 0.133 | 0.399 | 0.003 | 0.009 |
| TRAIL | 6.22±1.39 | 4.83±0.94 | 3.98±0.38 | ＜0.001 | ＜0.001 | ＜0.001 | ＜0.001 | 0.069 | 0.207 |
| Basic FGF | 5.01(4.81-5.64) | 5.37(4.96-5.75) | 4.03(4.03-4.05) | 0.126 | 0.378 | 0.001 | 0.002 | ＜0.001 | ＜0.001 |
| beta-NGF | 0.08(0-1.01) | 1.04(0-1.61) | 1.01±0 | 0.011 | 0.034 | 0.097 | 0.291 | 0.647 | 1.000 |
| HGF | 9.21±0.73 | 10.51±0.89 | 7.38±0.24 | ＜0.001 | ＜0.001 | ＜0.001 | ＜0.001 | ＜0.001 | ＜0.001 |
| PDGF-BB | 9.59(9.00-10.24) | 10.60(9.66-10.85) | 8.94±0.71 | 0.001 | 0.002 | 0.168 | 0.504 | 0.003 | 0.008 |
| VEGF | 0(0-0) | 0(0-4.33) | 5.37±0 | 0.589 | 1.000 | 0.001 | 0.003 | 0.002 | 0.007 |
| CTACK | 10.18(9.73-10.30) | 9.73±0.54 | 7.95±0.38 | 0.005 | 0.015 | ＜0.001 | ＜0.001 | 0.001 | 0.003 |
| Eotaxin | 6.67±0.72 | 6.36±0.58 | 5.67±0.33 | 0.023 | 0.070 | ＜0.001 | 0.002 | 0.014 | 0.042 |
| GRO-alpha | 10.34(9.47-10.73) | 10.91(9.53-10.36) | 10.36±0.20 | NS | NS | NS | NS | NS | NS |
| IP-10 | 111.98(11.10-12.74) | 12.54(11.89-14.08) | 7.62±0.29 | 0.008 | 0.024 | 0.002 | 0.006 | ＜0.001 | ＜0.001 |
| MCP-1 | 6.11±1.41 | 6.35±1.62 | 3.47±0.34 | 0.797 | 1.000 | ＜0.001 | 0.001 | ＜0.001 | 0.001 |
| MCP-3 | 1.40(0.65-2.91) | 2.68±1.18 | 0.19(0.19-0.20) | 0.001 | 0.002 | 0.011 | 0.033 | ＜0.001 | ＜0.001 |
| MIF | 9.94(9.32-10.42) | 10.24±0.75 | 5.86±0.23 | NS | NS | NS | NS | NS | NS |
| MIG | 9.58±1.20 | 11.71(11.00-12.44) | 5.86±0.53 | ＜0.001 | ＜0.001 | 0.014 | 0.042 | ＜0.001 | ＜0.001 |
| MIP-1alpha | 3.26(2.54-3.77) | 3.72±0.72 | 0.74±0.18 | 0.007 | 0.021 | 0.001 | 0.004 | ＜0.001 | ＜0.001 |
| MIP-1beta | 8.02(7.79-8.21) | 8.00(7.78-8.12) | 7.98±0.05 | NS | NS | NS | NS | NS | NS |
| RANTES | 11.91(11.58-12.39) | 11.81±0.87 | 12.23±0.25 | NS | NS | NS | NS | NS | NS |
| SDF-1alpha | 10.42±0.38 | 10.50±0.30 | 10.05±0.22 | 0.253 | 0.760 | 0.014 | 0.041 | 0.003 | 0.008 |

Notes: Cytokines were log2-transformed. Continuous variable data were presented as the mean (± SD) or the median (IQR). All p-values were adjusted using the Bonferroni correction or all pairwise comparison.
