# Supplementary material for: The differences in cytokine signatures between severe fever with thrombocytopenia syndrome (SFTS) and hemorrhagic fever with renal syndrome (HFRS)
Source: J Virol. 2024 Jun 25;98(7):e00786-24. doi: 10.1128/jvi.00786-24 (PMC11265425; doi:10.1128/jvi.00786-24)
Supplement: Table S3 — Statistically significant cytokines as determined by multiple linear regression analysis. [file jvi.00786-24-s0009.docx]

| Supplementary Table S3.Statistically significant cytokines as determined by multiple linear regression analysis | | | | | | |
| --- | --- | --- | --- | --- | --- | --- |
|  |  |  |  |  |  |  |
| SFTS vs. HFRS accounting for age and sex | | | | | | |
| cytokines | P*-*VALUE | Q-VALUE | EXP (COEF) | EXP (COEF)  95%CI | PERCENT (%)  DIFFERENCE | PERCENT (%)  DIFFERENCE 95%CI |
| IL-2Ralpha | ＜0.001 | ＜0.001 | 0.432 | (0.342, 0.547) | -56.8 | (-65.8, -45.3) |
| IL-8 | ＜0.001 | ＜0.001 | 0.326 | (0.239, 0.445) | -67.3 | (-76.1, -55.5) |
| IL-12(p40) | 0.003 | 0.008 | 0.582 | (0.408, 0.830) | -41.8 | (-59.2, -17.0) |
| IL-16 | 0.001 | 0.002 | 0.556 | (0.397, 0.781) | -44.4 | (-60.3, -21.9) |
| LIF | ＜0.001 | ＜0.001 | 0.587 | (0.454, 0.760) | -41.3 | (-54.6, -24.0) |
| GM-CSF | 0.014 | 0.028 | 0.707 | (0.537, 0.931) | -29.3 | (-46.3, -6.9) |
| SCF | 0.003 | 0.007 | 0.783 | (0.669, 0.918) | -21.7 | (-33.1, -8.2) |
| IFN-alpha 2 | 0.004 | 0.008 | 2.108 | (1.284, 3.462) | 110.8 | (28.4, 246.2) |
| TRAIL | ＜0.001 | ＜0.001 | 2.541 | (1.763, 3.663) | 154.1 | (76.3, 266.3) |
| HGF | ＜0.001 | ＜0.001 | 0.414 | (0.322, 0.532) | -58.6 | (-67.8, -46.8) |
| PDGFBB | 0.010 | 0.021 | 0.598 | (0.407, 0.880) | -40.2 | (-59.3, -12.0) |
| CTACK | 0.022 | 0.043 | 1.194 | (1.027, 1.388) | 19.4 | (2.7, 38.8) |
| MCP-3 | ＜0.001 | ＜0.001 | 0.450 | (0.309, 0.655) | -55.0 | (-69.1, -34.5) |
| MIG | ＜0.001 | ＜0.001 | 0.228 | (0.157, 0.331) | -77.2 | (-84.3, -66.9) |
| MIP-1alpha | 0.006 | 0.013 | 0.651 | (0.482, 0.880) | -34.9 | (-51.8, -12.0) |

Abbreviations: IL-2R alpha: Interleukin 2 receptor alpha, IL-8: Interleukin 8 IL-12(p40): Interleukin 12/P40, IL-16: Interleukin 16, LIF: Leukemia Inhibitory factor, GM-CSF: Granulocyte macrophage colony stimulating factor, SCF: Stem cell factor, IFN-alpha 2: Interferon alpha 2, TRAIL :TNF related apoptosis-inducing ligand, HGF: Hepatocyte growth factor, PDGF-BB: Platelet derived growth factor-BB , CTACK: Cutaneous T cell attracting chemokine, MCP-3: Monocyte chemoattractant protein 3, MIG: Monokine induced by gamma interferon, MIP-1alpha: Macrophage inflammatory protein 1 alpha, CI: Confidence interval.

P-values were adjusted using the Storey false discovery rate. EXP (COEF): Regression coefficient were transformed exponentially with base 2. PERCENT DIFFERENCE: Fold change was calculated by subtracting one from exponentiated model coefficients.
