# Supplementary material for: The differences in cytokine signatures between severe fever with thrombocytopenia syndrome (SFTS) and hemorrhagic fever with renal syndrome (HFRS)
Source: J Virol. 2024 Jun 25;98(7):e00786-24. doi: 10.1128/jvi.00786-24 (PMC11265425; doi:10.1128/jvi.00786-24)
Supplement: Table S4 — Predictive value of the top 10 cytokines extracted from the random forest model in predicting SFTS and HFRS. [file jvi.00786-24-s0010.docx]

| Supplementary Table S4. The predictive value of the top 10 cytokines extracted from the random forest in distinguishing SFTS and HFRS. | | | | | | |
| --- | --- | --- | --- | --- | --- | --- |
|  |  |  |  |  |  |  |
| Parameters | AUC | Cut off Values | Sensitivity | Specificity | 95%CI | *P* value |
| IL2Ralpha | 0.988 | 6.540 | 90 | 100 | (0.954,1.000) | ＜0.001 |
| IL8 | 1.000 | 4.612 | 100 | 100 | (1.000-1.000) | ＜0.001 |
| TRAIIL | 0.622 | 6.169 | 100 | 30 | (0.366-0.879) | 0.369 |
| MIG | 0.978 | 10.440 | 90 | 100 | (0.922-1.000) | ＜0.001 |
| IFNalpha2 | 0.556 | 2.982 | 70 | 55.56 | (0.285-0.827) | 0.683 |
| HGF | 0.922 | 9.607 | 90 | 88.89 | (0.787-1.000) | 0.0019 |
| MCP3 | 0.922 | 1.372 | 80 | 100 | (0.797-1.000) | 0.0019 |
| SCF | 0.789 | 6.587 | 70 | 88.89 | (0.570-1.000) | 0.034 |
| PDGFBB | 0.833 | 10.421 | 80 | 88.89 | (0.644-1.000) | 0.014 |
| CTACK | 0.689 | 10.073 | 60 | 88.89 | (0.440-0.938) | 0.165 |

Abbreviations: IL2Ralpha: Interleukin 2 receptor alpha, MIG: Monokine induced by gamma interferon, IFN alpha2: Interferon alpha 2, TRAIL: TNF related apoptosis-inducing ligand, HGF: Hepatocyte growth factor, IL 13: Interleukin 13, IL 8: Interleukin 8, PDGF-BB, Platelet derived growth factor-BB, IL 1beta: Interleukin 1beta G-CSF: Granulocyte colony stimulating factor.

The cut-off points were selected by maximizing the sum of sensitivity and specificity.
